# HGF/Met Signaling in Cancer Invasion: The Impact on Cytoskeleton Remodeling

**DOI:** 10.3390/cancers9050044

**Published:** 2017-05-05

**Authors:** Chuan Xiang, Junxia Chen, Panfeng Fu

**Affiliations:** 1 Department of Orthopedics, The Second Hospital of Shanxi Medical University, Taiyuan 030001, China; xcml7275@163.com; 2 Department of Cell Biology and Genetics, Chongqing Medical University, Chongqing 400016, China; chjunxia@126.com; 3 Department of Pharmacology, University of Illinois at Chicago, Chicago, IL 60612, USA

**Keywords:** hepatic growth factor (HGF), cancer, cytoskeleton, metastasis

## Abstract

The invasion of cancer cells into surrounding tissue and the vasculature is essential for tumor metastasis. Increasing evidence indicates that hepatocyte growth factor (HGF) induces cancer cell migration and invasion. A broad spectrum of mechanisms underlies cancer cell migration and invasion. Cytoskeletal reorganization is of central importance in the development of the phenotype of cancer cells with invasive behavior. Through their roles in cell mechanics, intracellular trafficking, and signaling, cytoskeleton proteins participate in all essential events leading to cell migration. HGF has been involved in cytoskeleton assembly and reorganization, and its role in regulating cytoskeleton dynamics is still expanding. This review summarizes our current understanding of the role of HGF in regulating cytoskeleton remodeling, distribution, and interactions.

## 1. Introduction

**Figure 1:**
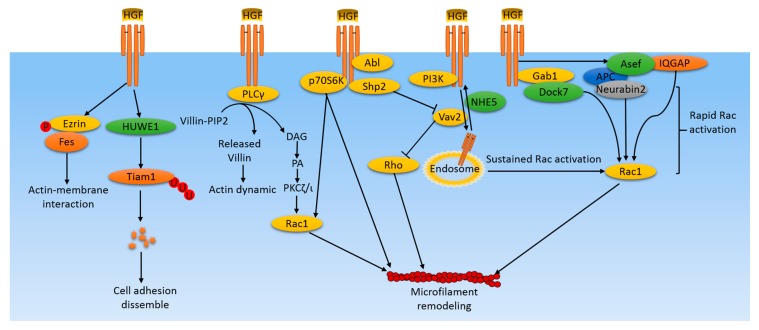
Microfilament-related pathways induced by HGF/cMet in cancer cells. HGF/cMet activates various pathways in cancer cells leading to microfilament remodeling, which plays an important role in cell lamellipodium formation, protrusion, migration, and metastasis. HGF induces rapid Rac activation by activating the Rac1 upstream regulator, GEF, for example, Asef and IQGAP. Simultaneously, Rac activity is maintained by distinct mechanisms involving the cMet receptor containing endosome translocations to the perinuclear area and to the cell membrane. In addition, some kinases and phosphatases are activated by HGF, leading to microfilament remodeling through the Rac/Rho system or through the direct interactions with actin filaments. Some supplemental mechanisms include ubiquitylization of signaling molecules, like Tiam, leading to perturbations of its downstream effectors, and phosphorylation of Ezrin, which mediates interactions between actin filaments and the cell membrane.

Hepatocyte growth factor (HGF), produced predominantly by mesenchymal cells, acts primarily through its only receptor, c-Met, in an endocrine and/or paracrine fashion [1]. It was originally identified as both a growth factor for hepatocytes and as a fibroblast-derived cell motility, or scatter, factor. A variety of cellular responses are activated by c-Met/HGF signaling. These responses mediate biological activities, including embryological development [2,3], wound healing [4,5], tissue regeneration [2,6], angiogenesis [7,8], invasion [9,10,11], and morphogenic differentiation [12]. As some of these physiological processes are important for tumor growth and metastasis, c-Met/HGF signaling has been identified as playing important roles in many human cancers. The mechanisms of c-Met/HGF signaling in regulating tumor growth and metastasis involve many aspects, including proliferation, angiogenesis in primary tumors, stimulating motility to form micrometastases, and branching morphogenesis [1]. Cancer cells gain uncontrolled ability to detach from the primary cancer colony and the capacity to migrate and invade. These changes in cellular morphogenesis and movement are the consequences of dramatic spatial and temporal reorganization of the cell cytoskeleton [13,14,15] (Figure 1). The cytoskeleton includes three major filaments—microfilaments, intermediate filaments, and microtubules—and proteins connecting cells and their extracellular matrix. In addition to these structural components, a variety of signaling molecules regulating cytoskeleton organization and remodeling are also targets activated in cancer cells. This review will focus on recent progress on the role of c-Met/HGF signaling in cytoskeleton protein dynamics and spacious rearrangements, and the related signaling molecules that are aberrantly activated, which lead to cancer migration and metastasis.

## 2. HGF and Microfilaments in Cancer

Microfilaments are composed of actin and actin-binding proteins. Actin exists either in monomeric (G-actin) or polymeric forms (F-actin). There are two isoforms of actin, the β- and γ-actins, which have been identified to exist in non-muscle cells. Cancer cells are characterized by dynamic reorganization of the actin cytoskeleton, which is critical for trans-differentiation of epithelial-like cells into motile mesenchymal-like cells, a process known as epithelial-mesenchymal transition (EMT) [15,16]. A reorganized actin cytoskeleton enables dynamic cell elongation and activated lamellipodial protrusions, where protrusive force is generated by the localized actin polymerization at the plasma membrane [17].

The organization and dynamics of the actin cytoskeleton are tightly regulated by the Rho family of small GTPases, in particular RhoA, Rac1, and Cdc42. RhoA regulates stress fibers and focal adhesions [18]. Rac1 regulates lamellipodia formation [19]. Cdc42 regulates the formation of filopodia and directional movement [20]. Therefore, HGF-regulated actin rearrangement is mainly through the regulation of small GTPase activity, but different types of cancer cells utilize distinct combinations of the signaling pathway in response to HGF to activate small GTPases. Small GTPases cycle between an inactive GDP-bound form and an active GTP-bound form. Activation of small GTPases is mediated by a family of 82 guanine nucleotide exchange factors (GEFs), while inactivation is promoted by a family of 67 GTPase-activating proteins (GAPs) [21]. Asef2, a GEF of Rac1 and Cdc42, is activated by HGF and forms a complex with neurabin2 and tumor suppressor adenomatous polyposis coili (APC) at lamellipodia and membrane ruffles to mediate HGF-induced cell migration [22]. Similarly, HGF-induced enhancement of the peripheral actin cytoskeleton in endothelial cells is dependent on Asef activity [23]. However, in this scenario, Asef forms a functional complex with the Rac1 effector, IQGAP1, and is localized at the cell cortical area in response to HGF stimulation [23]. Dock7 is another GEF of Rac. It was found to be elevated in human glioblastoma multiforme, a highly invasive primary brain tumor. HGF stimulation of glioblastoma resulted in interactions of Dock7 and c-Met in a manner that is dependent on the adaptor protein Gab1, which also interacts with Dock7 in an HGF-dependent manner [11].

HGF-induced Rac activation can also be through inhibition of guanine nucleotide dissociation inhibitor (GDI), which antagonizes both GEF and GAP and mediates the cycling of small GTPases between the cytosol and the membrane. HGF was shown to induce epithelial plasma membrane recruitment and activation of diacylglycerol kinase (DGK), which converts diacylglycerol (DAG) into phosphatidic acid (PA). PA generated at the plasma membrane then activates atypical PKCζ/ι, in complex with GDI and Rac. Then, Rac is released from the inhibitory complex with GDI, allowing its activation and formation of the actin cytoskeleton-driven membrane ruffles [24].

A recent study revealed a novel endosomal signaling mechanism linking c-Met/HGF signaling to the actin cytoskeleton via sustained Rac1 activation at the perinuclear endosome [25]. Despite an acute activation of Rac1 at the cell periphery, HGF induces c-Met trafficking to a perinulcear endosome for sustained activation of Rac1, which triggers optimal membrane ruffling, cell migration, and invasion. This sustained Rac1 activation needs PI3K/Vav2 activation. In contrast, another endosome-involved mechanism was proposed as a mechanism of persistent Rac1 activation. Unlike the requirement of c-Met trafficking to the perinuclear endosome for sustained Rac1 activation, this mechanism emphasizes the importance of the recycling of the c-Met-containing endosome to the plasma membrane for Rac1 persistent activation and actin cytoskeleton remodeling in C6 glioma cells. Neuron-enriched Na^+^/H^+^ exchanger NHE5 was identified as the mediator for endosomal recycling [26].

In addition to GEF and GDI, some kinases also mediate the actin cytoskeleton reorganization. The serine/threonine kinase, p70S6 kinase, is a downstream effecter of phosphatidylinositol 3-kinase/Akt pathway, which is activated by HGF in ovarian cancer [27]. Ectopic expression of constitutively-active p70S6K in ovarian cancer cells induces a marked reorganization of the actin cytoskeleton and promotes directional cell migration. p70S6 kinase was found to directly bind to, and cross-link with, actin filaments in response to HGF stimulation. p70S6 kinase does not affect actin polymerization, but can stabilize actin filaments by the inhibition of cofilin-induced actin depolymerization. In addition, p70S6K also mediates HGF-induced Rac1 and Cdc42 activation [27]. In our recent report, we found that HGF-induced actin cytoskeleton accumulation at the lamellipodia of endothelial cells is partially dependent on the intracellular sphingosine-1-phosphate (S1P) generation and efflux of S1P via its membrane transporter, Spns2, resulting in S1P receptor 1 activation, a process known as the “inside-out” signaling mechanism. Phospholipase Cγ (PLCγ) has long been recognized as downstream signaling molecule of c-Met/HGF [28]. PLCγ catalyzes the formation of inositole 1,4,5-trisphosphate (IP3) and diacylglycerol (DAG) from phosphatidylinositol 4,5-bisphosphate (PIP2), which regulates many actin-binding proteins [29]. HGF treatment of Madin-Darby canine kidney (MDCK) cells leads to PLCγ activation and subsequent generation of IP3 from PIP2 [30]. Villin, a member of the gelsolin family, binding to barbed ends of actin filaments and severing actin filaments was shown to bind to PIP2 at the cell membrane, thus inhibiting the capacity of villin to sever actin filaments [31]. Upon HGF stimulation of MDCK cells, a small pool of villin is phosphorylated and binds to activated PLCγ, thus increasing its PIP2-hydrolyzing activity and leading to IP3 production. The villin pool previously bound to PIP2 is released, and the villin-severing activity is promoted, leading to the actin cytoskeleton dynamics by generating new barbed ends at the plasma membrane at the leading edge of growing lamellipodia [32]. The Abl non-receptor tyrosine kinase regulates cytoskeletal dynamics through Rho family GTPases and actin regulatory proteins. It has been shown that HGF activates Abl in MDCK cells. Activated Abl kinase interacts with c-Met receptors and promotes its phosphorylation and downstream signaling pathways, including actomyosin contractility [33]. MAP4K4 is activated by HGF in medulloblastoma cells and is coupled to cortical actin polymerization and membrane protrusion [34].

As many cellular processes, including migration, are regulated by the balance of kinases and phosphatase, it is not surprising that phosphatases are also involved in HGF-induced actin cytoskeleton reorganization. In MDCK cells, dominant negative mutant of Shp2 inhibits HGF-induced cell scattering. This inhibitory effect of dominant negative mutants of Sph2 implies that HGF activates Shp2, which subsequently decreases Vav2 activity by dephosphorylation. Inhibition of Vav2, in turn, decreases Rho activity and leads to reduced actin stress fiber formation and focal adhesion formation [35]. However, activation of Rac in cancer cells promotes cell migration and invasion is not the case for some cancer cells, as cancer cells need to undergo cell-cell junction disassembly before they are ready for migration and, generally, Rac is considered to play a critical role in maintaining cell-cell adhesion junctions. Therefore, down-regulation of Rac activity and cell-cell adhesion junctions may be a driver for cell adhesion junction disassembly. In both MDCKII cells and lung cancer cells, HGF activates HECT, UBS, and WWE domain-containing protein 1 (HUWE1), a member of the HECT3 ubiquitin ligase family. Activated HUWE1 ubiquitylates Rac GEF, T lymphoma invasion, and metastasis-inducing protein 1 (Tiam1) on lysine 595, triggering its proteasomal degradation prominently at cell-cell adhesions, thereby enabling disassembly of cell junctions and the induction of cell migration and invasion [36].

Cell protrusion is the consequence of the interaction between cytoskeletons and the cell membrane. An emerging body of investigation has highlighted the role for Ezrin and the two closely related proteins radixin and moesin (ERM) in regulating the linkage between the membrane and the underlying cytoskeletons. Early studies have shown that Ezrin is the substrate of the c-Met receptor [37]. Phosphorylation of Ezrin tyrosine residue Y477 is important for HGF-induced cell scattering, which is blocked by Ezrin mutations at tyrosine residue Y477. Later, the non-receptor tyrosine kinase Fes has been identified as the effecter directly binding to the phosphorylated Y477 residue. Phosphorylated Ezrin recruits the Fes kinase at cell-cell contacts, where it becomes activated and is required for HGF-induced cell scattering [38].

Taken together, regulation of microfilaments by HGF in cancer cells relies on various mechanisms. Small GTPases play central roles in these signaling pathways. Most signaling pathways activated by HGF/Met converge on small GTPases through activation/inactivation of their regulators, GEF, GAP, and GDI. Pathways other than small GTPases also play important roles in actin cytoskeleton dynamics. Different cancer cells may utilize specific pathways for their migration and invasion (Figure 1).

## 3. HGF and Microtubule in Cancer

Like actin participation in cancer cell morphogenesis and metastasis, the microtubule system is also a major player in cancer cell migration and invasion [14,39]. Microtubules (MT) are composed of α- and β-tubulin heterodimers. Various α- and β-tubulin isotypes have been identified and display tissue- and developmental-specific expression. Microtubules play an important role in the segregation of chromosomes during mitosis [40]. Normally, the microtubule network is initiated from the centrosome and forms a hub for the formation of this network. When cells begin to divide, the microtubule network undergoes a complete remodeling to form the mitotic spindles. Upon completion of mitosis, the spindle is disassembled and the normal network reforms. The role of microtubules in cancer migration is attributed to its participation in cancer cell protrusion formation and the process of EMT [41]. Dynamic growth of MT is believe to mediate cell-directed migration and protrusion. Microtubule plus-end tracking proteins (+TIPs), a heterogeneous group of proteins that reversibly bind to growing MT ends, have been proposed to control MT dynamics and interactions with other intracellular structures [42]. The adenomatous polyposis coli protein (APC), spectraplakins (ACF7), and cytoplasmic linker-associated proteins (CLASPs) mediate MT interactions with cortical structures and signaling factors at the leading edge of migrating cells and may be important regulators of cell architecture [43,44,45]. Most +TIPs do not directly bind to growing MT ends, but are recruited by end-bind proteins (EBs), which are small dimeric proteins consisting of an N-terminal calponin homology domain that directly recognizes a structural feature of growing MT ends and a C-terminal EB homology domain that mediates localization of other +TIPs [46]. In the process of MDCK cell morphological changes in response to HGF, extensive reorganization of the MT cytoskeleton and MT dynamics were observed. EB1 and +TIPs were identified to be recruited to growing MT ends. Importantly, EB1 recruitment is required for MT reorganization as EB1 depletion disorganizes HGF-induced cell extensions and protrusion [47]. Additionally, EB1 has been identified to mediate HGF-induced Rac1 activation and assembly of the cortical actin cytoskeleton in endothelial cells, representing a mechanism for MT-regulated actin remodeling [48].

Tankyrase 2 is a poly(adenosine diphosphate)-ribose polymerase that is involved in the processes of Wnt signaling, telomere maintenance, vesicle trafficking, and microtube spindle pole assembly during mitosis. A very recent study showed that inhibition of Tankyase 2 hampers lung cancer cell invasion and migration in response to HGF, implicating that tankyase 2 mediates HGF-induced microtubule assembly in cancer cells. Mechanistically, the inhibitory effects of tankyase 2 blockade could be due to sequential deterioration of the distinct events that govern cell directional sensing. In particular, tankyase blockade negatively regulates microtubule dynamic instability, adenomatous polyposis coli plasma membrane targeting, and centrosome reorientation [49].

Crosstalk between the actin cytoskeleton and MT has been shown to be implicated in metastasis of cancer cells. For example, actin filaments that confer changes in cortical tension and contraction can be regulated by MT. Stathmin is a MT-associated regulatory protein [50]. Unphosphorylated stathmin destabilizes MT by reducing the MT polymer via sequestration of soluble tubulin into an assembly-incompetent T2S complex. Conversely, phosphorylation of stathmin reduces its MT-destabilization activity suggesting a cooperative nature of stathmin phosphorylation in control of MT depolymerization. HGF induces stathmin phosphorylation and increases the pool of stable MT in a Rac1-dependent manner. MT peripheral growth in ECs in response to HGF is mediated by stathmin phosphorylation [51]. Additionally, HGF also increases the peripheral microtubules and stimulates the growth of acetylated tubulin, which is an alternative mechanism of tubulin stabilization [51].

The role of MT in HGF-induced cancer cell migration and invasion may achieve, through its role as a mediator of signaling molecules, transportation from the cytosol to the cell membrane. There is evidence that HGF-induced lamellipodia formation of human breast cancer cells is dependent on WASP Verpolin homologous 2 (WAVE2), a member of the WASP/WAVE family of actin regulatory proteins, transporting from the cytosol to the leading edge membrane [52]. This process is mediated by MT, as MT depolymerization agent nocodazole inhibits WAVE2 transportation, suggesting the necessity of MT assembly for this transportation. Further, WAVE2 is found to interact with kinesin heavy chain KIF5B, one of the motor proteins. Thus, it is proposed that WAVE2 may be transported by KIF5B along MT to the leading edge of the lamellipodia in response to HGF. Another study also indicated the role of MT as transport mediators in the HGF-induced invasion of prostate cancer cells [53]. Similarly, this study also used nocodazole to disrupt MT. Imaging analysis revealed co-localization of MT and lysosomes at DU145 cell surface protrusions after HGF treatment. Pretreatment of cells with nocodazole abolished lysosome movement toward the cell surface. These results suggest that the MT-mediated location of lysosomes is an important aspect of cancer cell invasion.

**Figure 2:**
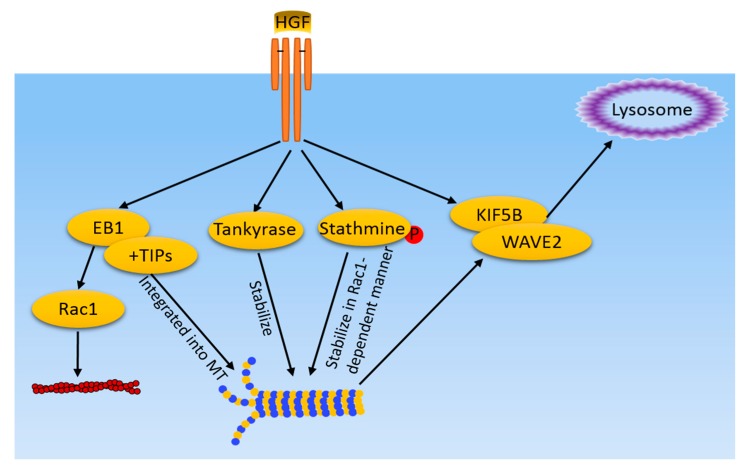
MT-related pathways induced by HGF/cMet in cancer cells. HGF/cMet activates multiple pathways, including EB1 and +TIPs recruitment to MT ends, tankyase, and stathmine-mediated stabilization of MT. Meanwhile, MT mediate WAVE2 and KIF5B translocation to the lamellipodia in response to HGF. Microfilaments and MT share a common regulator, EB1, which also regulates actin filament dynamics in a Rac1 dependent-manner.

Altogether, these data show that HGF-induced cell directional migration is mediated partially by the polarized MT network, which is characterized by differences along the directional migration, in MT plus-end dynamics, MT capture and anchoring at the cell cortex, and MT stability, which is regulated by MT-associated proteins and other cytoskeletal elements. MT are not only the targets of HGF-induced signaling pathways, but they also contribute to signaling transduction by MT-dependent transport of signaling molecules and apparatus (Figure 2).

## 4. HGF and Cell-Cell Junctions in Cancer

Cell-cell junctions are composed of a complex network of adhesion proteins that bind to each other and are linked to intracellular cytoskeletal and signaling partners. These proteins are organized into distinct structures and can be categorized into three groups, namely, adherens junctions (AJs), tight junctions (TJs), and gap junctions (GJs). They provide structural support for intercellular connection and maintain membrane integrity. An important step in the formation of cancer metastasis is the dissociation of cancer cells. HGF regulation of cancer cell metastasis may, through modulate cell-cell junctions’ stability and the expression of the component proteins. HGF modulates the levels of several TJ molecules’ (occludin, claudin-1, and 5, and JAM-1 and 2) mRNA transcripts in MDA MB 231 and MCF-7 cells. The mRNA data were confirmed by Western blot and immunohistochemistry staining [54]. HGF treatment of MDCK II cells leads to a loss of expression of claudin-2 in an Erk2-dependent manner [55]. HGF stimulation promotes phosphorylation of cell-cell junction proteins to regulate their stability at the junctions. Turnover of claudin-3 is greatly reduced after HGF stimulation in MDCK cells, which is believed to be mediated by its tyrosine phosphorylation sites in the C-terminal tail [56]. β-catenin was found to be phosphorylated at tyrosine residues in response to HGF. Phosphorylated β-catenin is dissociated from cell-cell junctions leading to a dismantling of cell-cell junction complexes. Prostate cancer cells have shown interactions between c-Met and E-cadherin/catenin complex after HGF stimulation [57]. In addition, immunofluorescence studies in the same cells revealed E-cadherin, β-catenin, and c-Met are co-localized at areas of cell-cell junctions after HGF stimulation. ZO-1 was found to have disappeared from the cell membrane after HGF treatment in primary mouse gastric epithelial cells. The dissociated ZO-1 is mediated by its tyrosine phosphorylation and is found in the cytoplasm and nucleus. However, HGF has no effect on ZO-1 expression [58]. Cell-cell junction molecules function in close cooperation with the actin cytoskeleton. HGF may regulate the junctional cytoskeleton to perturb junctional integrity. HGF reduces the junctional staining of myosine VI, which normally localizes to zonula adherence and interacts with E-cadherin in confluent Caco-2 cells [59]. Reduction of myosine VI leads to a dispersion of junctional actin filaments, which is responsible for the disruption of E-cadherin upon HGF stimulation. By contrast, findings from 3D collagen matrix cultures of the MDCK system make the relationship between cell-cell junctions and cytoskeleton in cancer cell migration more complicated [60]. Unlike the role of HGF in perturbing E-cadherin through actin cytoskeleton dispersion, cell-cell junctions are required for directional persistent collective cell migration in a 3D environment. In this case, HGF induces the actin cytoskeleton to align parallel to the cell-cell junctions and some actin bundles are organized perpendicular to the N-cadherin junctions in the direction of the contractile force generated by migrating cells. These changes in cytoskeletons allow cell clusters to migrate in a more persistent direction than single isolated cells, suggesting that cell-cell interactions between the leader cells and follower cells promote a linear migration path in the 3D matrix. One possible explanation for why cells prefer to migrate along, and with, adjacent cells is that the path provides the least resistance compared to the extracellular matrix.

The p21-activated kinases (PAKs) family has been implicated in the regulation of both cell matrix adhesion and migration, and they also play a role in junctional dynamics. PAK6 was identified as a putative IQGAP1 binding protein and its expression has been linked to prostate cancer invasiveness [57]. HGF-induced cell-cell dissociation has been linked to PAK6 activation in prostate cancer DU145 and colon cancer HT29 cells. HGF stimulation increases PAK6 autophosphorylation at S560 in both DU145 and HT29 cells. Downregulation of PAK6 by siRNA transfection reduces the scattering response of cells to HGF. A mechanistic study showed that PAK6 interacts with IQGAP1 through the C-terminus of PAK6 and the N-terminus of IQGAP, and this interaction is independent of Cdc42. Moreover, PAK6 can be immunoprecipitated with β-catenin and directly phosphorylate β-catenin at S675. Importantly, PAK6 and β-catenin are both localized at E-cadherin-positive cell junctions.

**Figure 3:**
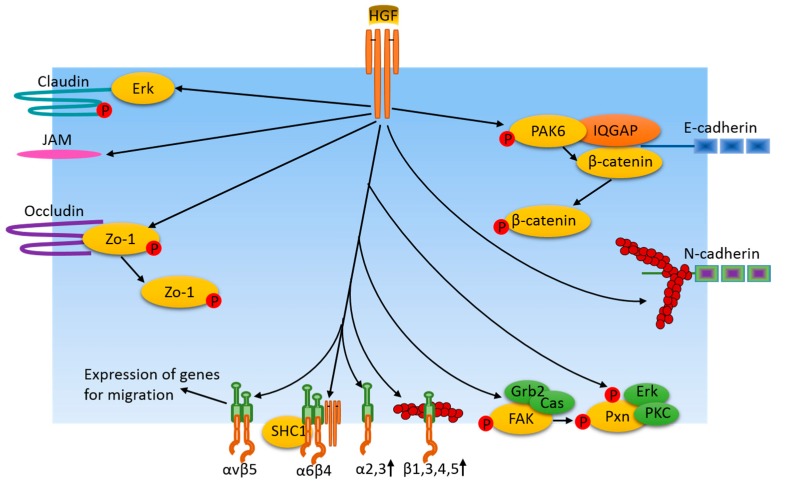
Cell adhesion junction and focal adhesion-related pathways induced by HGF/cMet in cancer cells. HGF regulates cell adhesion protein availability and adhesion junctions by means of protein expression or through activation of certain protein kinases which, in turn, phosphorylate adhesion junction proteins and their associated proteins. Additionally, HGF induces adhesion protein instability via the dispersion of junction area actin filaments. Similarly, HGF also regulates integrin levels at focal adhesions via their expression. In turn, integrins regulate the expression of genes for migration. FAK and paxillin are phosphorylated in response to HGF treatment, resulting in dynamic changes of focal adhesions, which are important for cancer cell motility.

Taken together, cell adhesions play a pivotal role in the development of cancer migration and metastasis. As shown in Figure 3, the major mechanism of cell adhesion-dependent regulation of cancer cell responses to HGF is achieved through the phosphorylation of cell adhesion molecules and their interacting molecules. Consequently, phosphorylated cell adhesion molecules become unstable at adhesion junctions and undergo dissociation from adhesion junctions. Therefore, cancer cells are activated for morphological change and motility.

## 5. HGF and Focal Adhesions in Cancer

Cells attach to their underlying matrices through complex transmembrane structures termed focal adhesions (FAs), which not only provide an anchor point for cells to adhere to the substratum, but also selectively recruit various signaling molecules to the sites, allowing cells to monitor their immediate environment. Formation and disassembly of FAs are regulated dynamically during cell migration. Cell adhesion to the extracellular matrix (ECM) is mainly mediated by the integrin family of cell-surface receptors. Integrins are composed of non-covalently linked α and β subunits, each of which is a transmembrane glycoprotein with a single membrane-spanning segment and, generally, a short cytoplasmic tail [61,62]. In humans, a total of 18 integrin α chains and eight β chains have been identified. They assemble in parallel arrays to form more than 24 heterodimers [62], each of which has a distinct, non-redundant function, binding to specific ECM components and soluble protein ligands. Integrin-ECM interaction initiates the occupancy and clustering of integrins and, in turn, promotes the recruitment of cytoskeletal and cytoplasmic proteins, such as talin, paxillin, and α-actinin to form focal complexes and focal adhesions [63]. In MDA-MB-231 cells, HGF signaling has been shown to selectively increase the adhesion to laminins 1 and 5, fibronectin, and vitronectin through a PI3 kinase-related mechanism [64]. HGF has been shown to regulate integrin avidity of β1, β3, β4, and β5. HGF triggers integrin clustering at actin-rich adhesive sites and lamellipodia [65]. Moreover, HGF also regulates the expression of some integrins. In MDCK cells, HGF treatment increases the expression of integrin α2 and, to a lesser extent, α3. Blocking of α2 by antibody blocks HGF-induced cell migration [66]. In highly-invasive mammary epithelial cells, the process of osteopontin-induced migration, which is dependent mainly on αvβ3 integrin, involves the activation of the HGF receptor. c-Met has been shown to form a complex with integrin α6β4, therefore, enhancing HGF-induced cancer cell invasion [67]. This effect may be due to the potency of HGF-induced Rac and PI3K signaling by α6β4-mediated recruitment of proteins, such as SHC1 and PI3K. Additionally, αvβ5 contributes to HGF signaling by controlling the expression of HGF-induced genes required for cell migration [68].

Focal adhesion kinase (FAK) is a critical non-receptor tyrosine kinase involved in the engagement of integrins and assembly of FA. It is significantly elevated in invasive and metastatic cancers, suggesting its role in cell migration [69]. FAK contains four principal regions mediating interactions with other adaptor and signaling proteins. The N-terminus contains a four-point-one, ezrin, radixin, moesin binding (FERM] domain, which acts as an autoinhibitory site by interacting with the kinase domain [70]. The catalytic tyrosine kinase domain is localized in the middle. At the C-terminus is the C-terminal focal adhesion targeting (FAT] domain providing additional sites for FAK binding partners that include paxillin and members of the Rho family proteins. Between FAT and the central catalytic domain is the proline-rich region, which provides binding sites for FAK binding partners along with the FAT domain [71]. Stimulation of small lung cancer cells with HGF activates c-Met and increases phosphorylation of Y397 of FAK. Within minutes after exposure of oral squamous cancer cells to HGF, FAK shows elevated tyrosine phosphorylation. Expression of FAK and HGF synergistically increases transformation of MDCK cells [72]. FAK mutants unable to bind PI3K or p130Cas do not promote the cell migration in response to HGF stimulation [66]. Additionally, FAK mutants defective in Grb2 binding migrate at an approximately 50% lower rate than wild-type FAK-expressing cells in response to HGF stimulation [66].

Paxillin is a 68 kDa scaffold protein with LIM1-4 domains at the C-terminus, LD1-5 domains at the N-terminus, and multiple tyrosine sites phosphorylated by FAK and Src kinases in response to growth factor stimuli and integrin-mediated cell-matrix adhesion. HGF has been shown to induce the phosphorylation of paxillin and matrix adhesion of prostate cancer cells, correlating with decreased matrix invasion [73]. The WASP Verpolin homologous 3 (WAVE3), a protein responsible for the regulation of actin polymerization through its interaction with Arp2/3, may mediate the phosphorylation of paxillin, but the underlying mechanism is unknown. In human hepatoma cell HepG2 cells, HGF induces both Ser178 and Tyr31 phosphorylation of paxillin, which regulates HepG2 migration. Phosphorylation of Ser178 is mediated by Erk kinase, and phosphorylation of Tyr31 is regulated by PKCε and ζ, although PKC kinase is a serine and threonine kinase. Interestingly, PKCα and δ downregulate Ser178 phosphorylation of paxillin through the suppression of Erk activity [74].

Overall, focal adhesion of cells to the ECM is key to the regulation of cellular morphology, migration, proliferation, and invasion. Distinct heterodimers of integrins are regulated by HGF at the level of expression in different cancer cells. Phosphorylation of FAK and paxillin in response to HGF plays an important role in transmitting signals from the HGF/cMet pathway during cell migration and invasion.

## 6. Conclusions

HGF/c-Met signaling has been intensively investigated for its role in cancer’s motility, invasion, metastasis, proliferation, and growth. Cytoskeleton remodeling and reorganization have been shown to be the major molecular mechanisms of HGF-induced cancer cell migration and metastasis. Different cancers may utilize distinct pathways triggered by HGF for migration and metastasis. Different cytoskeleton components interact with each other and share common upstream signaling pathways to cooperate and form a network to drive the metastasis of cancer cells. For example, crosstalk between the actin cytoskeleton and MT promotes a symmetry break to polarize cells for division, shape changes, and migration. MT depolymerization leads to actin stress fiber formation and actomyosin contraction. Furthermore, dissociation of actin cytoskeleton anchorage from cell-cell junctions facilitates the disruption of cell-cell junctions and, therefore, accelerates cancer cell scatter. Importantly, understanding the mechanisms that mediate HGF-induced cancer cell cytoskeleton dynamic reorganization has implications for the development of effective anti-metastasis cancer drugs.
